# Cross-Sectional and longitudinal associations of objectively-measured physical activity on blood pressure: evaluation in 37 countries

**DOI:** 10.15171/hpp.2017.34

**Published:** 2017-09-26

**Authors:** Mehdi Menai, Benoit Brouard, Matthieu Vegreville, Angela Chieh, Nicolas Schmidt, Jean-Michel Oppert, Hélène Lelong, Paul D. Loprinzi

**Affiliations:** ^1^Unaffiliated, Paris, France; ^2^Nokia Digital Health, Issy-les-Moulineaux, France; ^3^Department of Nutrition Pitié-Salpêtrière Hospital (AP-HP), Institute of Cardiometabolism and Nutrition (ICAN), Université Pierre et Marie Curie-Paris, Paris, France; ^4^Paris-Descartes University, Faculty of Medicine; Hôtel-Dieu Hospital; AP-HP; Diagnosis and Therapeutic Center, Paris, France; ^5^Department of Health, Exercise Science and Recreation Management, Physical Activity Epidemiology Laboratory, University of Mississippi, Mississippi, USA

**Keywords:** Epidemiology, Step counts, Blood pressure, Connected Objects

## Abstract

**Background:** We examined the cross-sectional and longitudinal associations of objectively-measured physical activity (step counts) and blood pressure (BP) among adults spanning 37 countries.

**Methods:** Across 37 countries, we used data from a pool of 9238 adult owners of Withings’ Pulse activity trackers, which measures steps taken each day, and Wireless Blood Pressure Monitor, which measures BP. Analyses were adjusted on age, sex, number of days where the tracker was worn, and number of BP measurements. Data was collected from 2009 to 2013.

**Results:** Subjects had a mean ± standard deviation (SD) age of 51.6 ± 11.3 years and a body mass index (BMI) of 28.7±5.5 kg/m^2^. A 1-month increase of more than 3000 steps per day was associated with a decrease of systolic BP (SBP) and diastolic BP (DBP) among the obese (1.57mm Hg and 1.29 mm Hg respectively, both P<0.001) and the overweight population (0.79 mm Hg and 0.84 mm Hg respectively, both P≤0.001), but not in the normal weight population (P=0.60 and P=0.36 respectively).

**Conclusion:** One-month increases in daily step counts was associated with a decrease of SBP and DBP in a large obese and overweight free living population.

## Introduction

Hypertension is a long-term medical condition in which the blood pressure (BP) in the arteries is persistently elevated. It is one of the most common risk factors for heart disease and stroke and the leading cause of mortality worldwide.^1^ Currently, it is estimated that 1 billion individuals globally are affected by hypertension, and projections indicate that 1.5 billion people will be affected by 2025.^1^ It has been shown to affect particularly obese populations,^2^ and treatments for hypertension add to the overwhelming medical burden due to obesity and cardiovascular disease worldwide.^3^ The World Health Organization (WHO) has noted that, among various interventions, promoting awareness in the general public about physical activity was successful in increasing physical activity behavior.^4^ Physical activity helps to manage non-communicable diseases such as hypertension but also cardiovascular disease, type 2 diabetes and certain cancers.^5^ Physical activity affects BP through various mechanisms including lowering vascular resistance or increasing insulin sensitivity.^6^ Encouragingly, research also demonstrates that physical activity may help to reduce premature mortality among those who are hypertensive^7^ and at risk for future cardiovascular disease.^8^ In addition to promoting physical activity, minimizing prolonged sedentary behavior, via intermittent walking, may be of critical importance as sedentary behavior is associated with an increased risk of future cardiovascular disease risk.^9^

Although numerous studies, including randomized controlled trials, have shown that general physical activity is associated with lower BP,^10^ studies for active commuting, and especially walking, are scarce. Randomized controlled trials have shown that walking tends to have beneficial effects on BP,^11^ but these studies include small samples and may not reflect practices in real life, which may lower the generalizability.^12^ To date, only a few studies involving large cohorts have investigated the association between free-living physical activity and BP, with the majority of these studies employing cross-sectional designs. Additionally, few of these studies have evaluated the association of free-living physical activity on ambulatory BP, despite ambulatory BP measurement being a better predictor of future cardiovascular disease when compared to clinic-based BP assessment.^13^

Several studies have shown that active transportation is associated with BP improvement or protection against hypertension,^14-16^ while others found no association.^17,18^ These mixed findings underscore the importance of future work on this topic. The practice of walking, specifically, was evaluated in only one study and was shown to associate with a lower prevalence of hypertension.^19^ Investigating the effects of walking on health outcomes is of critical importance as walking is the most common mode of physical activity among adults.^20^

To address these gaps in the literature, the purpose of the present study was to examine the cross-sectional and longitudinal associations of walking-based (via step counts), objectively-measured physical activity on BP among adults spanning 37 countries.

## Materials and Methods

### Study design and participants

This study examined the association of 1-month changes in physical activity on 1-month changes in BP. To address this, data were collected from users of the Withings’ Health Mate mobile application, a smartphone-based application launched in more than 100 countries in 2009 which tracks everyday activity levels using mobile phone sensors.

Data was collected from 2009 to 2013. Users were 18 years or older (age range 19–90 years). Among these participants, the study focused on users who had access to the Withings’ Pulse activity tracker (WPAT) and the Withings’ Wireless Blood Pressure Monitor (WWBPM). Physical activity data used is this study comes from the WPAT only, not the mobile phone sensors.

From this sample (n = 12 532), 1136 participants were excluded because of insufficient data for WPAT and 2750 for WWBPM (see below). Thus, our analytic sample included 9238 subjects with a mean ± standard deviation (SD) age of 51.6 ± 11.3 years. Among them, 30% were consumers from the United States, 22% from Germany, 8% from France, 5% from Japan and 5% from Switzerland (Table 1).

All users agreed to the anonymous use of their data when creating an account on the Withings’ Health Mate mobile application, which constitutes informed consent for this type of research.^21^ Withings complies with all privacy regulations in the countries where it operates (e.g. in Europe: Directive 95/46/CE and in the United States: Federal Trade Commission Act and the recommendations of the Federal Communications Commission). The mobile application, Health Mate, is available for free on the App Store and the Google Play Store, and it enables users to collect all data from their Withings’ devices in one single location. Users must have a smartphone or a tablet on iOS or Android to be able to download the application and to use the devices. Every user needs to create an account on the Health Mate mobile application to use Withings devices. This account includes information about users’ height, weight, age, and sex before they can use the WPAT and the WWBPM. Body mass index (BMI) was calculated as reported weight (kg) divided by reported height squared (m^2^) and categorized as normal (BMI <25 kg/m^2^), overweight (BMI ≥25 and <30 kg/m^2^) or obese (BMI ≥ 30 kg/m^2^).^22^

### Measures

*Withings’ Pulse activity tracker*

Step counts were assessed using the WPAT, an accelerometer-based device intended to be worn on the wrist as a watch, but can also be worn clipped on a belt or in a pocket. This type of device appears to be a reliable tool to report the number of steps taken.^23,24^ The WPAT uses a triaxial accelerometer to assess multi-plane movement. To ensure habitual movement patterns were assessed, only participants who had sufficient monitoring data were included in our analysis, which included having a minimum of 10 days of tracker wearing time, as defined by at least 500 steps a day, per month.^25^ The activity tracker does not require any action from the user to measure steps. We computed the daily mean step value of each month where the WPAT was used as well as the number of days per month for which the WPAT device was worn.

The change in steps recorded for 2 sequential validated months was categorized according to steps changes of 1000 to 3000 steps,^26^ recommendations for physical activity^27^ and data distribution (i.e. decrease of more than 3000 steps per day, decrease of between 3000 and 1000 steps per day, change between -1000 and +1000 steps per day, increase of between 1000 and 3000 steps per day and increase of more than 3000 steps per day). These thresholds have been selected based on prior recommendations of obtaining 1000 steps in ten minute walking bouts, or 300 steps in a 30 minute walking bout.^26^

*Withings’ Wireless Blood Pressure Monitor*

BP measurements were taken using the WWBPM that tracks systolic BP (SBP) and the number of BP measurements taken per day. The WWBPM has been validated for home BP measurement according to the European Society of Hypertension International Protocol.^28^ The WWBPM is a validated automatic device for home BP measurement at the arm level.^28^ Inflation is automatic and provided by an electric pump fitted on the cuff. Deflation is linear and actively controlled pressure release valve. SBP, diastolic BP (DBP), and pulse rate are displayed on the screen of the iOS or Android device. This device is available to the general public without any medical prescription and is able to collect data on a daily basis. It is intended to be used without medical supervision by the general population in their homes. Prior to taking a measurement, the mobile application shows the user a guide with good practices for BP measurement. Only those with a minimum of ten BP measurements recorded per month for days with WWBPM and WPAT records were analyzed herein. We computed the mean systolic and diastolic value of each day with at least 2 measurements and computed the mean value for the month and the change for 2 sequential validated months.

### Statistical analyses

Continuous variables were summarized by means ± SD and categorical variables by frequencies. Associations between daily BP (systolic or diastolic) and daily steps counts averaged by month were assessed using linear mixed models, which handle repeated data for each participant. We used quadratic regression to present graphic associations between BP and steps per day at the first month (which represent baseline, cross-sectional associations) (STATA 13; StataCorp LP, College Station, TX).

For the longitudinal analyses, we used data from the validated months (available BP measurement and step count as previously defined). The difference between mean values of BP (systolic or diastolic) for the validated months was used as the outcome variable. Similarly, the difference between mean values of the number of daily steps for each validated month was used as the independent variable. Sensitivity analyses demonstrated an interaction effect of physical activity and BMI on BP; thus, analyses were stratified according to weight status (normal weight, overweight and obesity). In all models, covariates included age, sex, number of days with recorded steps, and number of monthly BP measurements (per month and per day). Inclusion of time of day for the BP measurements as a covariate in the models did not appreciably alter the findings; thus, it was not included as a covariate in the model. For all analyses, the significance level was set at 0.05 and all tests were two-tailed. Non-graphical analyses were performed using SAS software (version 9.4, SAS Institute Inc., Cary, NC, USA).

## Results

### Characteristics of the study population

Subjects were mostly middle-aged (age range 19-90 years, mean between 50.7 and 52.6 years old across BMI group), with the large majority being men (Table 2). Subjects had validated data of WPAT and WWBPM for a mean of 4 months. BP was measured 2 times per day on average for days with at least one measurement. The obese population had a shorter follow-up, had less steps at baseline, and a higher SBP and DBP than other groups.

### Cross-sectional association between blood pressure and steps per day

**Figure 1:**
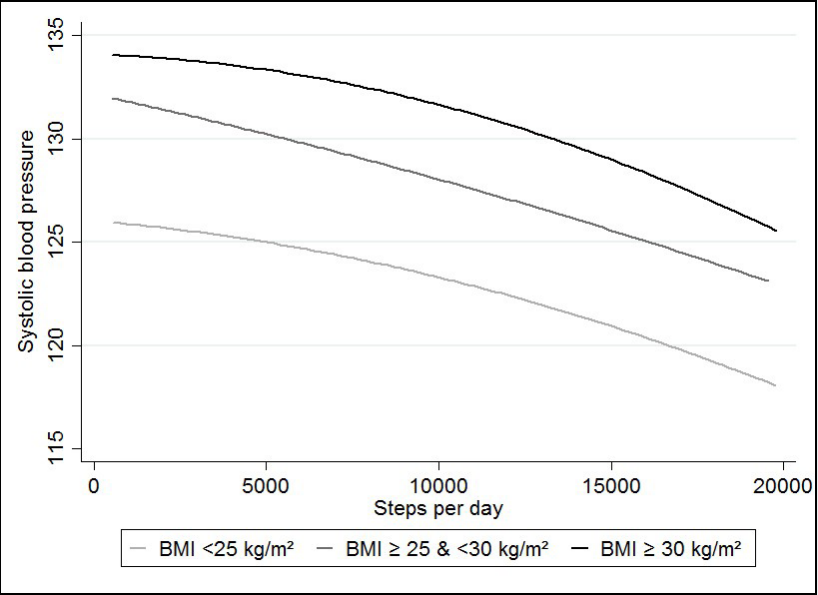
Association between steps/day and SBP, stratified by weight status‏.

**Figure 2:**
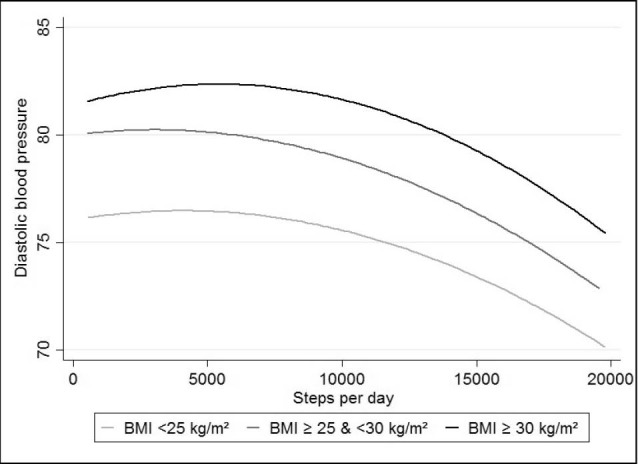
‏ Association between steps/day and DBP, stratified by weight status‏.

Analysis on the associations between SBP and mean steps per day highlighted a linear trend across BMI group, with differential intercepts (125.9, 132.0 and 134.4 mm Hg for normal weight, overweight, and obese subjects respectively, with fewer than 2500 steps per day) (Figure 1). In DBP analyses, graphic association showed a 2-part linear trend with differential intercepts (76.4, 80.2 and 82.7 mm Hg for normal weight, overweight, and obese subjects respectively, with less than 2500 steps per day) (Figure 2). The first part of the curve has a less marked slope compared to the second part, which starts between 8000 and 10 000 steps per day.

### Month-to-month longitudinal association between blood pressure and steps per day 

Longitudinal month-to-month analyses showed that all *P* values for trends were significant, reflecting a dose-response relationship between steps per day and BP (all *P*≤ 0.001) (Table 3). In the normal weight population, a decrease in steps per days between 3000 and 1000 was associated with an increase of DBP and SBP and increase of steps per day was not associated with BP levels. Such non-significant associations were not found in the overweight and obese populations. In these groups, an increase of more than 1000 steps per day was associated with a significant decrease of SBP and DBP, with the largest effect seen with an increase of more than 3000 steps per day (-1.57 mm Hg SBP and -1.29 mm Hg DBP in the obese group, *P* < 0.001).

### Sensitivity results 

The associations between step counts and BP in the overweight and obese groups were also observed using data with at least 15 and 20 days of physical activity tracker and BP measurements (Supplementary file 1). Additionally, in these analyses, an increase of more than 3000 steps per day was also found to be associated with a decrease of systolic BP in the normal weight group (-0.95 mm Hg, *P* = 0.01 and -0.89 mm Hg, *P* = 0.04 for 15 and 20 days/measurements, respectively).

## Discussion

In a large sample of middle-aged adults using Withings’ Pulse activity trackers and Wireless Blood Pressure Monitors, we observed an inverse cross-sectional association between mean daily step counts and mean daily systolic and diastolic BP. In addition, using longitudinal data, we found that a month-to-month increase in the number of steps per day was associated with significant lowering of both systolic and diastolic BP in overweight and obese populations.

Our results showed that objectively-measured step counts were associated with a lower BP. Existing literature in large cohorts commonly focuses on associations between indices of overall active transportation, mostly assessed subjectively, and objectively assessed BP or hypertension. Previous studies have found negative^15^ or no^17,18,29,30^ association between active transportation and hypertension or BP levels. For walking behavior specifically, Laverty et al found in 20 458 UK adult residents that when compared to driving, walking practices were negatively associated with the prevalence of self-reported hypertension (odds ratio [OR] = 0.83; 95% CI = 0.71; 0.97).^19^ While physical activity has been shown to prevent hypertension, the lack of significant results previously showed by other studies on BP may be due to imprecise measurements of active transportation, which may lead to a diminution of the apparent effects of physical activity on BP due to regression dilution bias.^31^

In our study, in addition to descriptive analyses, we performed a month-to-month assessment of changes in BP related to daily step counts. We found that an increase in daily step counts was associated with a short-term decrease of systolic and diastolic BP. The most similar study to ours was a study among US runners (n = 35 663), which highlighted that running distance was strongly negatively associated with incident hypertension for 7-8 years of follow-up.^32^ In intervention studies promoting walking, a meta-analysis of 26 studies has shown that participants who increased their average steps per day by approximately 2500 decreased their SBP by 3.8 mm Hg and their DBP by 0.3 mm Hg.^33^ Most of these pedometer-based interventions have focused on short-term changes in BP (4-16 weeks) as well as used a small sample of participants (total n = 468). The large amount of data that can be collected from this type of device may allow for sufficient statistical power to compute accurate predictors of associations between steps per day and BP. It may also help us to overcome the complexities of assessing short-term changes inherent to interventions, which have different lengths of follow-up and most of the time only 2 measurements (baseline and follow-up).

We also found that month-to-month changes in BP were apparently more pronounced in the obese population. A meta-analysis in 2002 showed that aerobic exercise was associated with lower levels of BP in 54 clinical trials in both normal and overweight subjects.^34^ A recent study highlighted in 10 339 participants that at a 3 year interval, obese women with a high physical activity level were less at risk of developing hypertension than obese inactive women (OR= 3.43, 95% CI = 2.68- 4.39; and OR = 4.91, 95% CI = 3.92-6.13, respectively).^35^ More recently, Crump et al showed in 93 035 men that there was an interaction between physical activity and BMI with incident hypertension over the mean 25.7 years of follow-up. They showed that fitness level was inversely associated with increased incidence of hypertension. This association was proportionally more pronounced in the normal weight group than for overweight/obese group.^36^ Our study confirms the interaction for a specific practice of physical activity (*as assessed by* step counts). However, we found that a short term association between daily step count and BP was more pronounced in overweight and obese populations. Differences between studies may be due to unmeasured confounding factors, different outcomes studied (hypertension and quantitative BP) or different lengths of follow-up. If confirmed and studied in further large cohort studies, these results in obese population could help to manage their BP with goals adapted to their precise weight and for various short term treatment durations.

Strengths of this study include the large study sample size across multiple countries, the assessment of cross-sectional and longitudinal relationships, and the objective assessment of step counts and BP. Limitations include the self-reporting of weight and height. Intensity of activity, length bouts and non-wear days were not assessed. Intensity of physical activity may affect BP through different mechanisms, but previous studies have shown that both light and moderate physical activity were found to lower ambulatory BP.^37,38^ Trained researchers did not monitor assessments of step counts and BP; however, our findings provide greater generalizability for the population. We lacked information on dietary factors and smoking status that may potentially modify associations between physical fitness or BMI and BP. More importantly, we did not have access to information on prior cardiovascular disease or medical treatment in this population. Physical activity, diet and smoking are known to be associated with each other.^39^ These behavioral factors are in turn associated with cardiovascular disease, from which high BP is a major risk factor. Finally, because our population was not representative of the general population, extrapolation of these findings must be done cautiously.

In conclusion, short term increase in daily step counts was associated with a decrease of SBP and DBP in obese and overweight owners of connected devices. This study reveals the link between short term month-to-month increase in step counts and lowered BP in overweight and obese populations, but need to be replicated in other population settings to increase the generalizability of these findings.

## Ethical approval

All procedures performed in studies involving human participants were in accordance with the ethical standards of the institutional and/or national research committee and with the 1964 Helsinki Declaration and its later amendments or comparable ethical standards. Informed consent was obtained from all individual participants included in the study. 

## Competing interests

MM, HL, JMO and PL declare no conflict of interest. BB, MV, AC and NS work for the company that provided the data.

## Authors’ contributions

MM performed analyses and wrote manuscript. HL and PL contributed discussion and reviewed/edited manuscript. JMO reviewed/edited manuscript. BB, MV, AC and NS researched data and reviewed/edited manuscript. All authors have read and approved the final version of the manuscript, and agree with the order of presentation of the authors.

**Table 1:** Countries of data collection

**Country**	**Frequency (%)**
United States	29.65
Germany	22.31
France	7.90
Japan	5.35
Switzerland	5.12
United Kingdom	4.87
Canada	2.98
Italy	2.31
Russian Federation	2.10
Austria	1.93
Estonia	1.74
Spain	1.47
Sweden	1.35
Australia	1.29
Belgium	1.02
Netherlands	0.87
Luxembourg	0.62
Czech Republic	0.62
Denmark	0.55
Poland	0.54
China	0.51
Norway	0.50
Hong Kong, SAR China	0.40
Brazil	0.36
Portugal	0.35
Ireland	0.30
Slovakia	0.28
Finland	0.25
Taiwan, Republic of China	0.25
Mexico	0.22
Bulgaria	0.12
New Zealand	0.11
Singapore	0.10
Greece	0.10
Korea (South)	0.10
Philippines	0.10
Others	1.14

**Table 2:** Characteristics of the study population (n = 9238)

	**Normal weight (n = 2235)**		**Overweight (n = 3903)**		**Obese (n = 3097)**	
	**Mean or %**	**SD**	**Mean or %**	**SD**	**Mean or %**	**SD**
Age	50.7	12.1	52.6	11.3	51.0	10.5
BMI	22.9	1.9	27.4	1.4	34.6	4.5
Sex (men)	82.6		92.6		91.5	
Length of follow-up (month)	4.1	3.0	3.9	2.8	3.6	2.5
Assessment of step counts per month						
Tracker worn (day)	22.9	9.1	22.5	9.0	21.9	8.9
Steps (per day)	7008	3664	6458	3250	5633	2983
Assessment of BP per month						
Number of measurements	31.2	25.6	32.8	33.7	31.6	26.7
Number of measurements per day^a^	2.3	1.7	2.5	1.9	2.4	1.7
Mean of SBP (mm Hg)	124.3	11.7	129.6	11.1	133.9	12.0
Mean of DBP (mm Hg)	75.9	8.3	79.7	8.4	83.5	8.8

Abbreviations: BP, blood pressure; SBP, systolic blood pressure; DBP, diastolic blood pressure; BMI, body mass index; SD, standard deviation. *P* for trend or χ^2^ were all <0.0001, except for the number of BP measurement per month (*P *= 0.80) and number of BP measurement per day (*P* = 0.01). ^a^ For days with at least one blood pressure measurement.

**Table 3:** Association of month-to-month changes in steps per day with month-to-month changes in blood pressure^a^

	**Normal weight (n = 2235)**		**Overweight (n = 3903)**		**Obese (n = 3097)**	
	***β***	***P***	***β***	***P***	***β***	***P***
Systolic blood pressure						
Decrease of more than 3000 steps per day	0.84	0.60	0.43	0.09	0.30	0.40
Decrease between 3000 and 1000 steps per day	**0.32**	**0.02**	0.08	0.54	0.17	0.31
Change between -1000 and 1000 steps per day	0 (Ref.)		0 (Ref.)		0 (Ref.)	
Increase between 1000 and 3000 steps per day	0.00	0.99	-0.20	0.14	**-0.33**	**0.05**
Increase of more than 3000 steps per day	-0.14	0.60	**-0.79**	**0.001**	**-1.57**	**<0.0001**
*P* for trend	**0.001**		**0.001**		**<0.0001**	
Diastolic blood pressure						
Decrease of more than 3000 steps per day	**0.54**	**0.002**	0.26	0.12	0.14	0.57
Decrease between 3000 and 1000 steps per day	**0.28**	**0.003**	0.13	0.15	**0.27**	**0.02**
Change between -1000 and 1000 steps per day	0 (Ref.)		0 (Ref.)		0 (Ref.)	
Increase between 1000 and 3000 steps per day	-0.05	0.65	**-0.20**	**0.02**	**-0.35**	**0.003**
Increase of more than 3000 steps per day	-0.16	0.36	**-0.84**	**<.0001**	**-1.29**	**<0.0001**
*P* for trend	**<0.0001**		**<0.0001**		**<0.0001**	

^a^Models were adjusted on age, sex, the number of day where the tracker was worn, monthly blood pressure measurements and daily blood pressure measurements for days with at least one blood pressure measurement. Bold text indicates statistical significance.

## Supplementary Materials

Supplementary file 1 contains Tables S1 and S2.
