# Supplementary material for: Cross-Sectional and longitudinal associations of objectively-measured physical activity on blood pressure: evaluation in 37 countries
Source: Health Promot Perspect. 2017 Sep 26;7(4):190–6. doi: 10.15171/hpp.2017.34 (PMC5647353; doi:10.15171/hpp.2017.34)
Supplement: Supplementary file 1 [file hpp-7-190-s01.pdf]

Health Promotion Perspectives, 2017, 7(4), 190-196
doi: 10.15171/hpp.2017.34

Table S1. Association of month-to-month changes in steps per day with month-to-month changes in blood pressure[a] using 15 wearing days per month of physical activity trackers and 15 blood pressure measurement per month

	**Normal weight (n=1,695)**		**Overweight (n=2,725)**		**Obese (n=1,917)**	
	**β**	***P***	**β**	***P***	**β**	***P***
Systolic blood pressure						
Decrease of more than 3,000 steps per day	0.48	0.20	0.59	0.11	0.46	0.41
Decrease between 3,000 and 1,000 steps per day	0.25	0.27	0.14	0.49	0.52	0.16
Change between -1,000 and 1,000 steps per day	0 (Ref.)		0 (Ref.)		0 (Ref.)	
Increase between 1,000 and 3,000 steps per day	-0.31	0.21	-0.77	0.001	-0.15	0.32
Increase of more than 3,000 steps per day	**-0.95**	**0.01**	**-1.37**	**<.0001**	**-1.43**	**0.004**
P for trend	**0.02**		**<.0001**		**0.0001**	
Diastolic blood pressure						
Decrease of more than 3,000 steps per day	0.39	0.10	0.42	0.09	0.30	0.41
Decrease between 3,000 and 1,000 steps per day	0.17	0.60	0.27	0.58	**0.58**	**0.003**
Change between -1,000 and 1,000 steps per day	0 (Ref.)		0 (Ref.)		0 (Ref.)	
Increase between 1,000 and 3,000 steps per day	-0.18	0.25	**-0.56**	**0.0001**	-0.27	0.16
Increase of more than 3,000 steps per day	**-0.62**	**0.01**	**-1.29**	**<.0001**	**-1.84**	**<.0001**
P for trend	**0.01**		**<.0001**		**<.0001**	

[a] Models were adjusted on age, sex, the number of day where the tracker was worn, monthly blood pressure measurements and daily blood pressure measurements for days with at least one blood pressure measurement

Table S2. Association of month-to-month changes in steps per day with month-to-month changes in blood pressure[a] using 20 wearing days per month of physical activity trackers and 20 blood pressure measurement per month

	**Normal weight (n=1,319)**		**Overweight (n=2,126)**		**Obese (n=1,416)**	
	**β**	***P***	**β**	***P***	**β**	***P***
Systolic blood pressure						
Decrease of more than 3,000 steps per day	0.49	0.25	0.70	0.11	**0.79**	**0.03**
Decrease between 3,000 and 1,000 steps per day	0.29	0.31	0.10	0.67	0.15	0.81
Change between -1,000 and 1,000 steps per day	0 (Ref.)		0 (Ref.)		0 (Ref.)	
Increase between 1,000 and 3,000 steps per day	-0.25	0.37	**-0.84**	**0.001**	0.22	0.77
Increase of more than 3,000 steps per day	**-0.89**	**0.04**	**-1.27**	**0.003**	-1.04	0.07
P for trend	0.05		**<.0001**		**0.04**	
Diastolic blood pressure						
Decrease of more than 3,000 steps per day	0.38	0.15	0.50	0.08	-0.08	0.87
Decrease between 3,000 and 1,000 steps per day	-0.03	0.87	0.05	0.76	0.32	0.22
Change between -1,000 and 1,000 steps per day	0 (Ref.)		0 (Ref.)		0 (Ref.)	
Increase between 1,000 and 3,000 steps per day	-0.24	0.18	**-0.61**	**0.0002**	-0.26	0.29
Increase of more than 3,000 steps per day	-0.51	0.07	**-1.30**	**<.0001**	**-1.43**	**0.0002**
P for trend	**0.01**		**<.0001**		**0.0001**	

[a] Models were adjusted on age, sex, the number of day where the tracker was worn, monthly blood pressure measurements and daily blood pressure measurements for days with at least one blood pressure measurement
